# Mental Health Differences in Healthcare Workers Exposed to Different Risks During the Coronavirus Disease 2019 Pandemic

**DOI:** 10.3389/fpsyt.2022.827076

**Published:** 2022-02-24

**Authors:** Yihui Zhang, Da Li, Xuan Ouyang, Hanping Bai, Linlin Zhao, Yongyan Shi, Liwen Tan

**Affiliations:** ^1^Department of Psychiatry, National Clinical Research Center for Mental Disorders, China National Technology Institute on Mental Disorders, The Second Xiangya Hospital of Central South University, Changsha, China; ^2^Institute of Anthropology, East China Normal University, Shanghai, China; ^3^Department of Psychiatry, Renmin Hospital of Wuhan University, Wuhan, China

**Keywords:** COVID-19, mental health, different risks, healthcare workers, China

## Abstract

**Background:**

The coronavirus disease 2019 (COVID-19) pandemic has caused a significant impact on the physical and mental health of healthcare workers. This study assessed the psychological status of healthcare workers who were exposed to different risk-levels in China and explored the factors that affected their mental health.

**Methods:**

Demographic, occupational characteristics, and mental health measurements were collected from 810 workers in 41 hospitals in China, through online questionnaires from February 11 to March 3, 2020. The degree of symptoms for fatigue, anxiety, and insomnia were assessed using the Chinese versions of the Fatigue Severity Scale, 7-item Generalized Anxiety Disorder Scale, and Insomnia Severity Index, respectively. Binary logistic regression analysis was performed to identify factors associated with mental health symptoms.

**Results:**

All 810 participants completed the relevant questionnaires without missing data. The prevalence of fatigue, anxiety, and insomnia symptoms was 74.3, 73.7, and 61.7%, respectively. Nurses, women, and workers exposed to high-risk areas were more likely to report mental health problems (*P* < 0.05). After controlling for confounders, exposure to high-risk areas was independently associated with increased symptoms of fatigue, anxiety, and insomnia (fatigue among high-risk areas: OR, 3.87; 95% CI, 2.26–6.61; *P* < 0.001; anxiety among high-risk areas: OR, 2.66; 95% CI, 1.58–4.51; *P* < 0.001; insomnia among high-risk areas: OR, 2.83; 95% CI, 1.68–4.79, *P* < 0.001).

**Conclusion:**

The study demonstrated significant differences in psychological symptoms among healthcare workers exposed to different levels of risk, and those in high-risk areas were more vulnerable to experiencing mental health symptoms. These findings emphasize the importance of giving due attention to healthcare workers, especially women, nurses, and those working in high-risk settings during the COVID-19 pandemic.

## Introduction

The coronavirus disease 2019 (COVID-19) pandemic has had devastating effects worldwide. Globally, as of October 29, 2021, there were 245,373,039 confirmed cases of COVID-19, including 4,979,421 deaths, reported to the WHO (1). As of October 29, 2021, there were 97,080 confirmed cases on the Chinese mainland, according to data from the National Health Commission (2).

The experience of previous pandemics has demonstrated that, in addition to the direct damage to physical functioning of the affected, emerging virus outbreaks also negatively impact mental health, particularly that of healthcare workers (3–5). The World Psychiatric Association has repeatedly issued statements calling attention to the mental health of healthcare workers (6). Previous studies on severe acute respiratory syndrome (SARS), Ebola, Middle East respiratory syndrome, and influenza A (H1N1) pandemic have revealed that public health emergencies significantly increase the workload of healthcare workers who face higher mortality rates and greater risks of infection than other communities due to the nature of their work; these factors can contribute to psychological problems among healthcare workers (7–10). Similarly, since the outbreak of COVID-19, studies on medical mental health have revealed that medical staff were particularly prone to anxiety (11, 12) and insomnia (13) at the early stage of the epidemic, with a higher prevalence of insomnia (14).

As per the experiences from previous pandemics, the exposure risks experienced by each healthcare worker vary, which may lead to differences in working hours, stress, and ultimately, differences in their mental health (15).

Over the course of the COVID-19 pandemic, the World Psychiatric Association has highlighted the different risks of exposure to healthcare workers (6). However, previous studies mostly investigated mental health problems of medical staff using regional or individual hospitals (13, 16) and did not analyze the mental health status of workers with different exposure risks in the early pandemic.

Therefore, we collected information on healthcare workers (doctors and nurses) who were exposed to varying levels of risk in China to explore the factors affecting their mental health in the early stage of COVID-19. Participants from different hospitals in Wuhan, Hubei province, and cities in other provinces were enrolled in this survey to analyze potential risk factors associated with symptoms such as fatigue, anxiety, and insomnia by quantifying the extent of these symptoms in participants and comparing differences in exposure to different levels of risk. The purpose of this study was to assess the psychological health of healthcare workers who were exposed to various risks in China, which may also serve as an effective evidence to guide the improvement of mental health of healthcare workers in various risk areas.

## Materials and Methods

### Participants and Procedures

This study is part of an investigation into mental health symptoms, associated risk factors, and relevant coping methods among healthcare workers across the country during the peak of COVID-19 in China. The 810 participants comprised staff (including 239 [29.5%] doctors and 571 [70.5%] nurses) from 41 hospitals during the early pandemic. Since Wuhan was the hardest-hit region, we sampled more hospitals in Wuhan, accounting for approximately three-quarter of the total sample size. This study was approved by the Clinical Research Ethics Committee of the Second Xiangya Hospital of Central South University.

The specific procedures were as follows. The survey was conducted on “Questionnaire Star,” a well-known online survey platform in China, from February 11 to March 3, 2020. The investigators distributed the link to the online questionnaire to workgroups at different hospitals through WeChat, spreading through a snowball sampling procedure (implying that each respondent was able to forward the link to another person). With informed consent, healthcare workers who received the link volunteered to participate in the study and could withdraw from the survey at any time. The online survey was anonymous and could only be completed once on the same device.

### Measures

#### Demographic and Occupational Characteristics Data

Demographic information mainly included gender (male or female) and age (18–25, 26–30, 31–40, 41–50, or 51–60). Occupational characteristics data primarily included occupation type (doctor or nurse), technical title (junior, intermediate, or senior), type of hospital (secondary or tertiary), location (Wuhan, Hubei province outside Wuhan, or other cities outside Hubei province), designated hospitals (yes or no), current position (fever clinic, mild ward, intensive care unit, medical technology, or logistics), and exposure risk (low, medium, or high). For evaluating exposure risk levels, participants were asked to answer four questions related to exposure risk. First, they were asked to state the risk level of their local area (selected by themselves after consulting the local government announcement), and the following questions were set considering the different opportunities for medical staff to come into contact with patients: whether the protective materials at their posts were sufficient; whether they were in a front-line position; and what level of exposure risk did they think individuals have at work? These questions more clearly instruct participants to identify their individual exposure risk level, rather than simply filling in the risk level for their location.

#### Fatigue, Anxiety, and Insomnia Symptoms

Previous studies have revealed that in the early stages of the outbreak, medical staff are prone to fatigue, anxiety, insomnia, and other acute symptoms, whereas depression is relatively insignificant (11–13). To reduce the efforts for answering questions and assess the mental health status of healthcare workers more efficiently and quickly, we assessed symptoms of fatigue, anxiety, and insomnia using the Chinese version of the standardized measurement tools (17–19). It includes three scales. First, the Fatigue Severity Scale (FSS) is used to assess fatigue symptoms and consists of nine items with a total score ranging from 0 to 63, with 36 or more being subjective fatigue (17). Second, the Generalized Anxiety Disorder-7 (GAD-7) assesses the severity of anxiety over the past 2 weeks, which contains seven items with a total score ranging 0–21 (18). The relationship between total scores and severity was as follows: normal (0–4), mild (5–9), moderate (10–14), and severe (15–21) anxiety. Lastly, the Insomnia Severity Index (ISI), a self-report tool that assesses the intensity of insomnia during the previous 2 weeks, contains seven items with a total score ranging 0–28 (19). The corresponding relationship between the total score and severity of insomnia was normal (0–7), mild (8–14), moderate (15–21), and severe (22–28) insomnia.

### Statistical Analysis

SPSS statistical software (version 26.0; IBM Corp) was used for data analysis. The ranked data are shown as numbers and percentages, calculated from the scores of each level for symptoms of fatigue, anxiety, and insomnia. To assess the intensity of each symptom between two or more groups, the non-parametric Mann–Whitney *U*-test and Kruskal–Wallis test were used. Binary logistic regression analysis was used to identify potential risk factors for fatigue, anxiety, and insomnia symptoms in participants, and the associations between risk factors and outcomes were presented as odds ratios (ORs) and 95% confidence intervals (CIs), after controlling for confounders such as gender, age, occupation type, technical title, type of hospital, location, departments, designated hospital, current position, and exposure risk. Statistical significance was set at *P* < 0.05.

## Results

In the study, 810 healthcare professionals completed the survey, of whom 239 (29.5%) were doctors and 571 (70.5%) were nurses. Among the respondents, 577 (71.2%) were exposed to high-risk areas, 163 (20.1%) were exposed to medium-risk areas, and 70 (8.6%) were exposed to low-risk areas. The primary distribution of the respondents across gender, age, job title, affiliated hospital, and risk area was as follows: women (662 [81.7%]), 26–40 years old (505 [62.4%]), junior technical title (422 [52.1%]), working in a tertiary hospital (537 [66.3%]), working in a designated hospital (622 [76.8%]), and belonging to medium or high-risk areas (740 [91.3%]; Table 1).

**Table 1:** Demographic and occupational characteristics of participants.

**Characteristic**	**Total (%)**	**Occupation**		**Exposure risk**		
		**Physician**	**Nurse**	**Low**	**Medium**	**High**
	810 (100.0)	239 (29.5)	571 (70.5)	70 (8.6)	163 (20.1)	577 (71.2)
**Gender**						
Male	148 (18.3)	129 (54.0)	19 (3.3)	25 (35.7)	27 (16.6)	96 (16.6)
Female	662 (81.7)	110 (46.0)	552 (96.7)	45 (64.3)	136 (83.4)	481 (83.4)
**Ages (years)**						
18–25	119 (14.7)	7 (2.9)	112 (19.6)	8 (11.4)	18 (11)	93 (16.1)
26–30	217 (26.8)	26 (10.9)	191 (33.5)	14 (20.0)	41 (25.2)	162 (28.1)
31–40	288 (35.6)	116 (48.5)	172 (30.1)	27 (38.6)	69 (42.3)	192 (33.3)
41–50	149 (18.4)	72 (30.1)	77 (13.5)	16 (22.9)	30 (18.4)	103 (17.9)
51–60	37 (4.6)	18 (7.5)	19 (3.3)	5 (7.1)	5 (3.1)	27 (4.7)
**Technical title**						
Junior	422 (52.1)	56 (23.4)	366 (64.1)	30 (42.9)	69 (42.3)	323 (56.0)
Intermediate	315 (38.9)	125 (52.3)	190 (33.3)	28 (40.0)	78 (47.9)	209 (36.2)
Senior	73 (9.0)	58 (24.3)	15 (2.6)	12 (17.1)	16 (9.8)	45 (7.8)
**Type of hospital**						
Secondary	273 (33.7)	100 (41.8)	173 (30.3)	31 (44.3)	69 (42.3)	173 (30.0)
Tertiary	537 (66.3)	139 (58.2)	398 (69.7)	39 (55.7)	94 (57.7)	404 (70.0)
**Location**						
Wuhan	615 (75.9)	129 (54.0)	486 (85.1)	45 (64.3)	104 (63.8)	466 (80.8)
Hubei province	62 (7.7)	21 (8.8)	41 (7.2)	7 (10.0)	14 (8.6)	41 (7.1)
Outside	133 (16.4)	89 (37.2)	44 (7.7)	18 (25.7)	45 (27.6)	70 (12.1)
**Designated hospitals**						
Yes	622 (76.8)	147 (61.5)	475 (83.2)	44 (62.9)	113 (69.3)	465 (80.6)
No	188 (23.2)	92 (38.5)	96 (16.8)	26 (37.1)	50 (30.7)	112 (19.4)
**Current position**						
Fever clinic	64 (7.9)	25 (10.5)	39 (6.8)	4 (5.7)	10 (6.1)	50 (8.7)
Mild ward	358 (44.2)	87 (36.4)	271 (47.5)	43 (61.4)	74 (45.4)	241 (41.8)
Intensive care unit	247 (30.5)	27 (11.3)	220 (38.5)	5 (7.1)	29 (17.8)	213 (36.9)
Medical technology	102 (12.6)	85 (35.6)	17 (3.0)	12 (17.1)	37 (22.7)	53 (9.2)
Logistics department	39 (4.8)	15 (6.3)	24 (4.2)	6 (8.6)	13 (8.0)	20 (3.5)

The severity categories of fatigue, anxiety, and insomnia were measured in the total cohort and subgroups. Most of the participants had subjective fatigue (602 [74.3%]), anxiety (597 [73.7%]), and insomnia symptoms (500 [61.7%]). Compared with physicians, nurses were more likely to report severe symptoms of anxiety (86 [15.1] vs. 30 [12.6], *P* = 0.024) and insomnia (42 [7.4] vs. 12 [5.0], *P* = 0.001). Compared with men, women were more likely to report fatigue (505 [76.3%] vs. 97 [65.5%], *P* = 0.007), severe anxiety (97 [14.7%] vs. 19 [12.8%], *P* = 0.021), and moderate insomnia (123 [18.6%] vs. 17 [11.5%], *P* = 0.006). The healthcare workers who reported exposure to high-risk areas were more likely to experience fatigue, severe anxiety, and severe insomnia than those exposed to medium- and low-risk areas (fatigue: 462 [80.1%] vs. 106 [65.0%], and 34 [48.6%], *P* < 0.001; severe anxiety: 102 [17.7%] vs. 11 [6.7%], and 3 [4.3%], *P* < 0.001; and severe insomnia: 47 [8.1%] vs. 5 [3.1%], and 2 [2.9%], *P* < 0.001). Compared with those working in non-designated hospitals, participants working in COVID-19 designated hospitals were more likely to report symptoms of fatigue (476 [76.5%] vs. 126 [67.0%], *P* = 0.009) and severe insomnia (47 [7.6%] vs. 7 [3.7%], *P* < 0.001). Compared with healthcare workers in “Hubei outside Wuhan” and “outside Hubei,” healthcare workers in Wuhan were more likely to report symptoms of fatigue (472 [76.7%] vs. 36 [58.1%], and 94 [70.7%], *P* < 0.001), anxiety (94 [15.3%] vs. 10 [16.1%], and 12 [9.0%], *P* < 0.001), and insomnia (94 [15.3%] vs. 10 [16.1%], and 12 [9.0%], *P* < 0.001; Tables 2.1, 2.2).

**Table 2.1:** Severity categories of fatigue, anxiety, and insomnia measurements in total cohort and subgroups.

**Severity** **category**	**Total, No (%)**	**Occupation**		**Z**	***P***	**Gender**		**Z**	***P***	**Ages (years)**					**H**	***P***
		**Physician**	**Nurse**			**Male**	**Female**			**18–25**	**26–30**	**31–40**	**41–50**	**51–60**		
**FSS**																
No.fatigue	208 (25.7)	69 (28.9)	139 (24.3)	−1.344	0.179	51 (34.5)	157 (23.7)	−2.703	0.007	24 (20.2)	48 (22.1)	75 (26.0)	49 (32.9)	12 (32.4)	5.178	0.270
Fatigue	602 (74.3)	170 (71.1)	432 (75.7)			97 (65.5)	505 (76.3)			95 (79.8)	169 (77.9)	213 (74.0)	100 (67.1)	25 (67.6)		
**GAD-7**																
Normal	213 (26.3)	76 (31.8)	137 (24)	−2.257	0.024	49 (33.1)	164 (24.8)	−2.303	0.021	30 (25.2)	47 (21.7)	88 (30.6)	41 (27.5)	7 (18.9)	7.315	0.120
Mild	311 (38.4)	89 (37.2)	222 (38.9)			58 (39.2)	253 (38.2)			48 (40.3)	86 (39.6)	109 (37.8)	59 (39.6)	9 (24.3)		
Moderate	170 (21.0)	44 (18.4)	126 (22.1)			22 (14.9)	148 (22.4)			21 (17.6)	48 (22.1)	56 (19.4)	31 (20.8)	14 (37.8)		
Severe	116 (14.3)	30 (12.6)	86 (15.1)			19 (12.8)	97 (14.7)			20 (16.8)	36 (16.6)	35 (12.2)	18 (12.1)	7 (18.9)		
**ISI**																
Normal	310 (38.3)	111 (46.4)	199 (34.9)	−3.273	0.001	72 (48.6)	238 (36)	−2.727	0.006	47 (39.5)	76 (35)	116 (40.3)	58 (38.9)	13 (35.1)	4.974	0.290
Mild	306 (37.7)	84 (35.1)	222 (38.9)			49 (33.1)	257 (38.8)			45 (37.8)	85 (39.2)	107 (37.2)	58 (38.9)	11 (29.7)		
Moderate	140 (17.3)	32 (13.4)	108 (18.9)			17 (11.5)	123 (18.6)			21 (17.6)	42 (19.4)	48 (16.7)	21 (14.1)	8 (21.6)		
Severe	54 (6.7)	12 (5.0)	42 (7.4)			10 (6.8)	44 (6.6)			6 (5)	14 (6.5)	17 (5.9)	12 (8.1)	5 (13.5)		

*FSS, fatigue severity scale; GAD-7, 7-item generalized anxiety disorder; ISI, 7-item insomnia severity index*.

**Table 2.2:** Severity Categories of Fatigue, Anxiety, and Insomnia Measurements in Subgroups.

**Severity** **category**	**Exposure risk**			**H**	***P***	**Designated hospitals**		**Z**	***P***	**Location**			**H**	***P***
	**Low**	**Medium**	**High**			**Yes**	**No**			**Wuhan**	**Hubei province**	**Outside Hubei**		
**FSS**														
No.fatigue	36 (51.4)	57 (35.0)	115 (19.9)	52.478	0.000	146 (23.5)	62 (33.0)	−2.613	0.009	143 (23.3)	26 (41.9)	39 (29.3)	19.653	0.000
Fatigue	34 (48.6)	106 (65.0)	462 (80.1)			476 (76.5)	126 (67.0)			472 (76.7)	36 (58.1)	94 (70.7)		
**GAD-7**														
Normal	33 (47.1)	50 (30.7)	130 (22.5)	49.875	0.000	152 (24.4)	61 (32.4)	−1.569	0.117	152 (24.7)	19 (30.6)	42 (31.6)	13.726	0.001
Mild	23 (32.9)	76 (46.6)	212 (36.7)			246 (39.5)	65 (34.6)			235 (38.2)	23 (37.1)	53 (39.8)		
Moderate	11 (15.7)	26 (16.0)	133 (23.1)			134 (21.5)	36 (19.1)			134 (21.8)	10 (16.1)	26 (19.5)		
Severe	3 (4.3)	11 (6.7)	102 (17.7)			90 (14.5)	26 (13.8)			94 (15.3)	10 (16.1)	12 (9.0)		
**ISI**														
Normal	43 (61.4)	79 (48.5)	188 (32.6)	44.507	0.000	220 (35.4)	90 (47.9)	−3.657	0.000	220 (35.8)	30 (48.4)	60 (45.1)	12.130	0.002
Mild	19 (27.1)	61 (37.4)	226 (39.2)			237 (38.1)	69 (36.7)			231 (37.6)	25 (40.3)	50 (37.6)		
Moderate	6 (8.6)	18 (11.0)	116 (20.1)			118 (19.0)	22 (11.7)			115 (18.7)	6 (9.7)	19 (14.3)		
Severe	2 (2.9)	5 (3.1)	47 (8.1)			47 (7.6)	7 (3.7)			49 (8.0)	1 (1.6)	4 (3.0)		

*FSS, fatigue severity scale; GAD-7, 7-item generalized anxiety disorder; ISI, 7-item insomnia severity index*.

For all participants, the median (IQR) scores on the FSS, the GAD-7, and the ISI scales were 44.0 (35.0–53.0), 7.0 (4.0–12.0), and 9.0 (5.0–14.0), respectively. Similarly, nurses, women, individuals exposed to high-risk areas, and those working in COVID-19 designated hospitals in Wuhan had higher scores on all scales. Specifically, among all participants, nurses scored higher than doctors on fatigue (46.0 [36.0–54.0] vs. 42.0 [33.0–48.0], *P* < 0.001), anxiety (7.0 [5.0–12.0] vs. 7.0 [4.0–11.0], *P* = 0.004), and insomnia (10.0 [6.0–15.0] vs. 8.0 [4.0–13.0], *P* < 0.001) symptom scales. Women were more likely than men to report high scores for fatigue (45.0 [36.0–54.0] vs. 40.0 [30.3–49.0], *P* < 0.001), anxiety (7.0 [5.0–12.0] vs. 6.0 [2.0–10.0], *P* = 0.001), and insomnia (10.0 [6.0–15.0] vs. 8.0 [3.0–13.0], *P* = 0.001) symptom scales (Table 3.1). Compared with those exposed to medium- and low-risk areas, participants who were exposed to high-risk areas reported higher scores in the three scales (fatigue: 46.0 [38.0–55.0] vs. 40.0 [31.0–51.0], 35.0 [26.8–43.0]; anxiety: 8.0 [5.0–13.0] vs. 6.0 [3.0–9.0], 5.0 [1.0–7.0]; and insomnia: 10.0 [6.0–15.0] vs. 8.0 [4.0–12.0], 6.0 [1.8–9.0]; *P* < 0.001). Moreover, participants working in a designated hospital reported higher scores than those working in a non-designated hospital (fatigue: 42.0 [32.0–49.0] vs. 45.0 [36.0–54.0], *P* < 0.001; anxiety: 7.0 [5.0–12.0] vs. 6.0 [2.0–11.0], *P* = 0.001; and insomnia: 10.0 [6.0–15.0] vs. 8.0 [3.3–12.0], *P* < 0.001). Similarly, compared to the participants in other cities of Hubei province (other than Wuhan) and other provinces, those in Wuhan reported higher scores in the three scales (fatigue: 45.0 [36.0–54.0] vs. 37.5 [27.5–47.3] and 43.0 [34.0–49.0], *P* < 0.001; anxiety: 7.0 [5.0–12.0] vs. 5.5 [2.0–9.3] and 6.0 [3.5–11.0], *P* = 0.001; and insomnia: 10.0 [6.0–15.0] vs. 8.0 [4.0–11.3] and 8.0 [4.0–13.0], *P* = 0.002; Table 3.2).

**Table 3.1:** Scores of fatigue, anxiety, and insomnia measurements in total cohort and subgroups.

**Scale**	**Total score median (IQR)**	**Occupation (IQR)**		**Z**	***P***	**Gender (IQR)**		**Z**	***P***	**Ages (years) (IQR)**					**H**	***P***
		**Physician**	**Nurse**			**Male**	**Female**			**18–25**	**26–30**	**31–40**	**41–50**	**51–60**		
FSS (fatigue)	44.0 (35.0–53.0)	42.0 (33.0–48.0)	46.0 (36.0–54.0)	−3.909	0.000	40.0 (30.3–49.0)	45.0 (36.0–54.0)	−3.633	0.000	43.0 (37.0–54.0)	47.0 (37.0–54.0)	43.0 (35.0–50.8)	44.0 (31.0–55.0)	49.0 (23.0–57.0)	5.178	0.270
GAD-7 (anxiety)	7.0 (4.0–12.0)	7.0 (4.0–11.0)	7.0 (5.0–12.0)	−2.846	0.004	6.0 (2.0–10.0)	7 (5.0–12.0)	−3.287	0.001	7.0 (4.0–10.0)	7.0 (5.0–12.0)	7.0 (4.0–11.0)	7.0 (4.0–12.0)	11.0 (4.5–17.0)	7.315	0.120
ISI (insomnia)	9.0 (5.0–14.0)	8.0 (4.0–13.0)	10.0 (6.0–15.0)	−3.958	0.000	8.0 (3.0–13.0)	10.0 (6.0–15.0)	−3.375	0.001	10.0 (6.0–14.0)	10.0 (6.0–15.0)	9.0 (5.0–14.0)	8.0 (4.0–14.0)	11.0 (5.0–17.0)	4.974	0.290

*FSS, fatigue severity scale; GAD-7, 7-item generalized anxiety disorder; ISI, 7-item insomnia severity index*.

**Table 3.2:** Scores of fatigue, anxiety, and insomnia measurements in subgroups.

**Scale**	**Exposure risk**			**H**	***P***	**Designated hospitals**		**Z**	***P***	**Location**			**H**	***P***
	**Low**	**Medium**	**High**			**Yes**	**No**			**Wuhan**	**Hubei province**	**Outside Hubei**		
FSS (fatigue)	35.0 (26.8–43.0)	40.0 (31.0–51.0)	46.0 (38.0–55.0)	52.478	0.000	45.0 (36.0–54.0)	42.0 (32.0–49.0)	−3.825	0.000	45.0 (36.0–54.0)	37.5 (27.5–47.3)	43.0 (34.0–49.0)	19.653	0.000
GAD-7 (anxiety)	5.0 (1.0–7.0)	6.0 (3.0–9.0)	8.0 (5.0–13.0)	49.875	0.000	7.0 (5.0–12.0)	6.0 (2.0–11.0)	−3.226	0.001	7.0 (5.0–12.0)	5.5 (2.0–9.3)	6.0 (3.5–11.0)	13.726	0.001
ISI (insomnia)	6.0 (1.8–9.0)	8.0 (4.0–12.0)	10.0 (6.0–15.0)	44.507	0.000	10.0 (6.0–15.0)	8.0 (3.3–12.0)	−4.155	0.000	10.0 (6.0–15.0)	8.0 (4.0–11.3)	8.0 (4.0–13.0)	12.13	0.002

*FSS, fatigue severity scale; GAD-7, 7-item generalized anxiety disorder; ISI, 7-item insomnia severity index*.

After controlling for confounding factors, binary logistic regression analysis revealed that nurses and women were more susceptible to fatigue (fatigue among nurses: OR, 0.54; 95% CI, 0.32–0.91; *P* = 0.022; fatigue among women: OR, 1.83, 95% CI, 1.07–3.14, *P* = 0.028). Compared with working in a secondary hospital, working in a tertiary hospital was associated with increased anxiety symptoms (OR, 1.45; 95% CI, 1.03–2.05; *P* = 0.032). Exposure to medium-risk areas was associated with increased anxiety symptoms than exposure to low-risk areas (OR, 1.91; 95% Cl, 1.06–3.45; *P* = 0.031). Compared with working in a non-designated hospital, working in a COVID-19 designated hospitals was associated with increased symptoms of insomnia (OR, 1.37; 95% CI, 0.95–1.97, *P* = 0.090). Exposure to high-risk areas was associated with increased fatigue, anxiety, and insomnia symptoms (fatigue: OR, 3.87; 95% CI, 2.26–6.61; *P* < 0.001; anxiety: OR, 2.66; 95% CI, 1.58–4.51; *P* < 0.001; and insomnia: OR, 2.83; 95% CI, 1.68–4.792; *P* < 0.001; Table 4).

**Table 4:** Risk factors for mental health symptoms identified by binary logistic regression.

**Variable**	**No. of symptomatic cases/** **No. of total cases (%)**	**Adjusted OR** **(95%CI)**	***P*** **-value^a^**
FSS, fatigue symptoms	629/859 (73.2)		
**Occupation**						
Physician	170/239 (71.1)	Reference	
Nurse	432/571 (75.7)	0.54 (0.32–0.91)	0.022
**Gender**						
Male	97/148 (65.5)	Reference	
Female	505/662 (76.3)	1.83 (1.07–3.14)	0.028
**Exposure risk**						
Low	34/70 (48.6)	Reference	
Medium	106/163 (65.0)	1.73 (0.96–3.12)	0.068
High	462/577 (80.1)	3.87 (2.26–6.61)	0.000
GAD-7, anxiety symptoms	621/859 (72.3)		
**Occupation**						
Physician	163/239 (68.2)	Reference	
Nurse	434/571 (76.0)	0.98 (0.6–1.6)	0.927
**Gender**						
Male	99/148 (66.9)	Reference	
Female	498/662 (75.2)	1.22 (0.73–2.06)	0.444
**Type of hospital**						
Secondary	188/273 (68.9)	Reference	
Tertiary	414/537 (77.1)	1.45 (1.03–2.05)	0.032
**Exposure risk**						
low	37/70 (52.9)	Reference	
Medium	113/163 (69.3)	1.91 (1.06–3.45)	0.031
High	447/577 (77.5)	2.66 (1.58–4.51)	0.000
ISI, insomnia symptoms	519/859 (60.4)		
**Occupation**						
Physician	128/239 (53.6)	Reference	
Nurse	372/571 (65.1)	1.1 (0.7–1.72)	0.689
**Gender**						
Male	76/148 (51.4)	Reference	
Female	424/662 (64.0)	1.42 (0.88–2.29)	0.149
**Designated hospitals**						
Yes	402/622 (64.6)	1.37 (0.95–1.97)	
No	98/188 (52.1)	Reference	0.090
**Exposure risk**						
Low	27/70 (38.6)	Reference	
Medium	84/163 (51.5)	1.55 (0.87–2.77)	0.140
High	389/577 (67.4)	2.83 (1.68–4.79)	0.000

*FSS, fatigue severity scale; GAD-7, 7-item generalized anxiety disorder; ISI, 7-item insomnia severity index; OR, odds ratio*.

a *P-value for each category vs. the reference*.

## Discussion

This study assessed the mental health of healthcare workers exposed to different risks in the early stages of the COVID-19 pandemic and analyzed the risk factors. In 2022, the global pandemic and the epidemic in China have once again reached a severe state, and the results of our study may strengthen the government's early attention to the mental health of medical staff and provide more perspectives and evidence for psychological prevention and intervening measure of healthcare workers. In this survey, a total of 810 healthcare professionals who were exposed to different risks in China received and completed all questions in the online questionnaire. All participants were divided into three groups based on their exposure risk: low-risk areas (70), medium-risk areas (163), and high-risk areas (577), and interregional differences were compared. Our results showed that most of the participants had mental health concerns, with symptoms of fatigue (74.3%), anxiety (73.7%), and insomnia (61.7%). In addition, nurses, women, those working in tertiary hospitals, in COVID-19 designated hospitals, in Wuhan, and those exposed to medium-and high-risk areas were more likely to exhibit symptoms pointing to mental health concerns. In all aspects of interest, exposure to high-risk areas was an independent risk factor for poor mental health.

Thus, the results of this study highlight that more attention should be given to the mental health of healthcare workers who reported exposure to high-risk environments.

This study indicated that a significant proportion of healthcare workers had fatigue symptoms, but the rate was significantly higher than in one study of frontline nurses in Wuhan that reported 35.06% of respondents having fatigue (20). Moreover, a previous study conducted early in the SARS outbreak in Taiwan, China, showed that 77.4% of respondents reported anxiety and worry, 52.3% experienced sleep problems, and obvious anxiety symptoms were more prominent in the initial stage (7). Therefore, the results of our study are consistent with those of previous studies. However, compared with studies among healthcare workers during the COVID-19 pandemic in China and Italy, our study showed higher rates of anxiety (13, 21–23). Furthermore, the percentage of healthcare workers with insomnia in our study was higher than the pooled prevalence of sleep disorders in Chinese healthcare professionals during the COVID-19 pandemic, which was ~45.1% (24). In the early pandemic period, longer working hours, a lack of protective equipment and supplies (25), and quarantine of self from family by healthcare workers significantly increased their perceived level of risk and psychological stress, which may exacerbate their daytime fatigue and affect mood and sleep patterns (14, 26, 27). In addition, the reasons why our study observed a higher rate of fatigue, anxiety, and insomnia may be due to differences in sample sources and sampling time. In this study, 71.2% of the participants worked in high-risk environments and may experience more work-related stress. Moreover, we discovered some differences in mental health between doctors and nurses, with nurses being more likely to experience fatigue, anxiety, and insomnia symptoms in the early pandemic period. Similarly, studies from other countries have revealed that the mental health burden on healthcare workers during the COVID-19 pandemic is significantly higher among nurses than doctors, owing to a greater exposure to both patients' and families' suffering and distress (28, 29).

More importantly, this survey showed that there were considerable disparities in the prevalence of fatigue, anxiety, and insomnia symptoms among healthcare professionals of different genders, with women scoring significantly higher than men. Similar results were also reported in a recent study of gender differences in mental health among healthcare workers during the COVID-19 pandemic (30). Moreover, it was consistent with earlier studies on healthcare professionals and the general public in the early stages of the SARS (7, 31) and COVID-19 pandemic (32, 33). Epidemiological studies have revealed that the lifetime prevalence of anxiety disorders is ~1.5-times higher in women than in men (34, 35), and adult women had significantly higher rates of insomnia reported (36–38). These differences may be influenced by many physiological factors, such as sex chromosome genes, sex hormones, and the activity of the hypothalamic-pituitary-adrenal axis (39, 40). Additionally, under significant stress, women tend to adopt more emotion-centered coping styles, such as complaint, avoidance, and self-blame, which are associated with increased symptoms of anxiety and insomnia (34).

What makes the study more remarkable is that it discovered that health care professionals at different exposure risks had different rates of mental health symptoms, with those in high-risk areas more likely to experience fatigue, anxiety, and insomnia. Moreover, it is an independent risk factor for poor mental health. Studies in China, Poland, Italy, Switzerland, and other countries during the COVID-19 pandemic have also revealed that front-line workers, especially those in high-risk areas, such as the intensive care unit, the infectious diseases unit, and the emergency departments, were at a much higher risk of anxiety, insomnia, and depressive symptoms than second-line workers (15, 41–43). This result may be due to the following reasons. First, the workload of front-line staff is overwhelming, especially during the outbreak of COVID-19, and the number of infections has increased sharply, and staff are more prone to fatigue symptoms, anxiety, and other psychological problems (44–47). Second, with regard to psychological aspects, compared with the staff in low-risk areas, the staff exposed to the high-risk setting and having a direct contact with a significant number of infected people, may have greater concerns about exposure to infection coupled with the patient's negative emotions, protective material shortage, lack of contact with family, and guilt from not being able to save each patient, leading to a significant increase in the psychological pressure of frontline staff (20, 26). Research has revealed that psychological stress is directly related to mental health problems such as anxiety and insomnia, and the greater the psychological stress, the higher the likelihood of these symptoms (45, 48, 49). The European Psychiatric Association also issued a statement highlighting the necessity of paying attention to psychological problems and early intervention among frontline workers (50). In addition, our study suggests that governments can allocate the number of mental health workers and the corresponding treatment model according to the level of exposure risk to more accurately maintain the mental health of healthcare workers and achieve a more effective use of resources.

### Study Limitations

This study had some limitations. First, our study focused on risk factors affecting the mental health of healthcare workers and lacked analysis of protective factors during the pandemic. Recent research has argued that resilience, emotion regulation, and social support may play a protective role in healthcare workers during the pandemic, and these protective factors may be operating to grant healthcare workers the necessary resilience in facing the enormous challenges posed by the pandemic (51). Second, the study was cross-sectional, reflecting only the mental health of healthcare professionals at that point in time, and could not reflect causality. Third, all data were collected through self-reported questionnaires, which may have increased the recall bias. Moreover, a convenience sampling method was adopted to recruit participants, which may limit the representativeness of the samples and the generality of the research results to some extent. Finally, our survey did not consider respondents' prior mental and physical conditions, which may have had some impact on their reported results.

In conclusion, the study of healthcare workers revealed a significant prevalence of fatigue, anxiety, and insomnia, especially among women during the COVID-19 pandemic. The survey results also demonstrated significant differences in psychological symptoms among healthcare workers exposed to different levels of risk, in which those in high-risk areas are more vulnerable to experiencing mental health symptoms. These findings remind us to pay more attention to healthcare workers, especially women and nurses, and those working in high-risk settings during the COVID-19 pandemic, while providing them with more support, including medical material support, personnel support, family support, and as early as possible to carry on psychological intervention, to maintain their mental health.

## Data Availability Statement

The raw data supporting the conclusions of this article will be made available by the authors, without undue reservation.

## Ethics Statement

The studies involving human participants were reviewed and approved by the Clinical Research Ethics Committee of the Second Xiangya Hospital of Central South University. The patients/participants provided their written informed consent to participate in this study. Written informed consent was obtained from the individual(s) for the publication of any potentially identifiable images or data included in this article.

## Author Contributions

LT, HB, DL, and XO contributed to the study concept and design. LZ and YS acquired and collected the data. YZ analyzed the data and drafted the manuscript. All authors read and approved the final manuscript.

## Funding

This work was supported by grants from the National Natural Science Foundation of Hunan Province, China (2020JJ4807).

## Conflict of Interest

The authors declare that the research was conducted in the absence of any commercial or financial relationships that could be construed as a potential conflict of interest.

## Publisher's Note

All claims expressed in this article are solely those of the authors and do not necessarily represent those of their affiliated organizations, or those of the publisher, the editors and the reviewers. Any product that may be evaluated in this article, or claim that may be made by its manufacturer, is not guaranteed or endorsed by the publisher.
